# The Hospitals, Etc., of New York

**Published:** 1859-10

**Authors:** 


### EDITORIAL DEPARTMENT.

### *The Hospitals, etc., of New York.*

Bellevue Hospital.—The great asylum for the sick poor of New York, stands at the head of the medical charities of the city, and as a field for the clinical study of disease is especially pre-eminent.

Situated almost in the very heart of the metropolis, its gates are open day and night to the almost hourly entrance of carts, carriages, or litters, bearing to its numerous wards disease and injury in all their myriad forms; while its happy location, directly upon the East river, opens still another avenue of access, where every day at 12 o'clock the almshouse steamer contributes its score of patients. Within fifteen minutes' walk of any of the medical colleges, its tiers of crowded wards are daily thrown open for clinical observation, and the student or practitioner can hardly fail in the course of a single visit to meet with abundance of material, either for general study or special investigation.

The hospital is situated upon First Avenue, and occupies with its grounds and out-buildings six acres of land, included between 26th and 28th Streets, the avenue and the river. It is a massive stone building of four stories, constructed in the form of an imperfect letter H. Two broad wings stretch east and west, one a length of 150, the other of 300 feet, connected at their middle, and inclosing between their free extremities on either side the tasteful grounds of the institution. The river-front of the building is furnished along each story with an iron-framed verandah for the benefit of convalescents. The central building, with a front of 240 feet, has an additional story, surmounted by a rotunda. In this rotunda is the operating theatre, which, with its ample accommodations for an audience of five hundred persons, is twice a week open for lecture and demonstration. Here in the course of a single season may be witnessed, perhaps I may say, every operation for the relief of surgical disease or injury that science can suggest or skill perform. Sometimes, also, it is used for the illustration of remarks upon such medical disease as admits of a collection of its cases from the various wards of the house. It is, however, in these wards, and by the bedside, that the more constant and substantial opportunities are offered to the student. Medical, surgical, and obstetrical inclusive, the wards number 37; of these, 24 are medical, 9 surgical, while 4 are embraced in the lying-in department. The first two classes are arranged in seven " divisions," four medical, three surgical; the former comprising a certain number for general cases, a " fever-ward " for the exclusive reception of typhus and typhoid fever, and a range of six " cells " for delirium tremens. Such is the arrangement both on the male and female side, except that with the latter is included the obstetrical department, consisting of a " waiting," a " lying-in," and a " convalescent " ward. Among the surgical the only ward having an especial office is that devoted to ulcers. With these few exceptions, the diseases are not classified.

Bellevue is intended especially for acute disease; small-pox is alone excluded. The average number of patients is one thousand; one thousand and three hundred can be accommodated; eight thousand are treated here yearly.

The attending medical staff of the hospital comprises eight physicians and eight surgeons. Two of the former and three of the latter are always in daily attendance. Two consecutive months is the usual length of each one's term of service.

The house-staff consists of fourteen recent graduates; four house physicians, three house-surgeons, and seven assistants. Each house-physician or surgeon has the care of one division, while each in turn takes charge of the obstetrical department for one month. The incumbents of these posts are appointed by the medical board. For this purpose semi-annual examinations are held, which are open to all who may choose to become candidates.

Clinical instruction is given on nearly every day in the week by those in attendance. They are accompanied by the class to the bedside of each patient, where, beside the remarks of the lecturer, the fullest opportunity is offered the student for personal investigation of individual cases.

In the south-east corner of the hospital grounds stands the " Pathological Building " so called; a two-story edifice of brick, recently erected with a view especially of facilitating the study of pathology and anatomy. On the ground-floor is a well-appointed and commodious autopsy-room where post-mortem examinations are held in the presence of students, and the appearances thus found pointed out and commented upon. On the floor above is a large, well-lighted apartment designed and used as a lecture-room and museum. Seats for two hundred and fifty persons are so arranged as to command a view of the demonstrator's table, while around the sides are glazed cases, containing the constantly increasing collection of morbid specimens found in the room below, well preserved and mounted, and having a history of the case attached to each.

Through the winter months regular courses of lectures are here given on Saturday afternoons, when the medical colleges are closed. These lectures are most fully illustrated with the material supplied by the mortality of the hospital. Surgical anatomy and midwifery have thus far claimed the principal share of attention in this department.

As a still further incentive to zeal among the students of the profession in the city, certain gentlemen of the medical board have established "Prizes." These prizes are awarded for superior excellence in dissection and anatomical preparation. The lists are open to all undergraduates in the several schools of the city; and, although established but a few years since, the cases of the museum already most brilliantly attest the favor with which the plan has been received, and the good results it has accomplished.

Such are the prominent features in this great school of medical science. The thousand minor advantages can only be appreciated by a personal familiarity.

